# Infection with *Salmonella enterica* Serovar Typhimurium Leads to Increased Proportions of F4/80^+^ Red Pulp Macrophages and Decreased Proportions of B and T Lymphocytes in the Spleen

**DOI:** 10.1371/journal.pone.0130092

**Published:** 2015-06-12

**Authors:** Kristin L. Rosche, Alanoud T. Aljasham, James N. Kipfer, Bryan T. Piatkowski, Vjollca Konjufca

**Affiliations:** 1 Department of Microbiology, Southern Illinois University, Carbondale, Illinois, United States of America; 2 Department of Plant Biology, Southern Illinois University, Carbondale, Illinois, United States of America; Université Libre de Bruxelles, BELGIUM

## Abstract

Infection of mice with *Salmonella enterica* serovar Typhimurium (*Salmonella*) causes systemic inflammatory disease and enlargement of the spleen (splenomegaly). Splenomegaly has been attributed to a general increase in the numbers of phagocytes, lymphocytes, as well as to the expansion of immature CD71^+^Ter119^+^ reticulocytes. The spleen is important for recycling senescent red blood cells (RBCs) and for the capture and eradication of blood-borne pathogens. Conservation of splenic tissue architecture, comprised of the white pulp (WP), marginal zone (MZ), and red pulp (RP) is essential for initiation of adaptive immune responses to captured pathogens. Using flow cytometry and four color immunofluorescence microscopy (IFM), we show that *Salmonella*-induced splenomegaly is characterized by drastic alterations of the splenic tissue architecture and cell population proportions, as well as in situ cell distributions. A major cause of splenomegaly appears to be the significant increase in immature RBC precursors and F4/80^+^ macrophages that are important for recycling of heme-associated iron. In contrast, the proportions of B220^+^, CD4^+^ and CD8^+^ lymphocytes, as well as MZ MOMA^+^ macrophages decrease significantly as infection progresses. Spleen tissue sections show visible tears and significantly altered tissue architecture with F4/80^+^ macrophages and RBCs expanding beyond the RP and taking over most of the spleen tissue. Additionally, F4/80^+^ macrophages actively phagocytose not only RBCs, but also lymphocytes, indicating that they may contribute to declining lymphocyte proportions during *Salmonella* infection. Understanding how these alterations of spleen microarchitecture impact the generation of adaptive immune responses to *Salmonella* has implications for understanding *Salmonella* pathogenesis and for the design of more effective *Salmonella*-based vaccines.

## Introduction

*Salmonella* spp. are Gram-negative facultative intracellular pathogens that infect their hosts via contaminated food and water. *Salmonella enterica* serovar Typhi infects humans, while *Salmonella enterica* serovar Typhimurium (*Salmonella*) in addition to humans, infects a variety of other species including rodents, cattle, and poultry [1, 2]. Infection of mice with *Salmonella* causes a disease that is similar to human typhoid, thus it is a widely used model of *S*. *typhi* infection [3]. The pathology observed during murine salmonellosis is complex and is characterized by general inflammation, fever, anemia, and splenomegaly [4, 5]. The infection begins by invasion of the intestines, from where *Salmonella* spreads to deeper tissues such as the mesenteric lymph nodes, the liver, and the spleen by CD18-expressing phagocytes [6]. As infection progresses, *Salmonella* causes systemic inflammation that is mediated largely by lipopolysaccharide (LPS) and TLR4 signaling which leads to renal tissue hypoxia [5, 7]. The reduction of available oxygen is sensed in the kidneys, which respond by increasing the synthesis of erythropoietin (EPO) [8]. EPO stimulates red blood cell (RBC) precursors to transition through a series of erythroblasts, eventually shedding their nuclei and fully differentiating into mature RBCs [9]. The expression of CD71 (a transferrin receptor) is high in RBC precursors and diminishes as the precursors mature into RBCs, while the expression of erythroid-specific cell marker Ter119 increases as RBCs mature [10]. EPO exerts its effect on developing erythroid cells in the bone marrow to stimulate erythropoiesis and counteract the tissue hypoxia [9, 11]. However, in spite of increased EPO production during *Salmonella* infection [5], the proportions of RBCs in systemic circulation decline leading to anemia [4]. Inefficient erythropoiesis during *Salmonella* infection stimulates splenic extramedullary erythropoiesis, resulting in the accumulation of immature RBC precursors and leukocytes [5, 12]. The spleen is a lymphoid organ in which old and damaged RBCs are phagocytosed, iron is recycled, and where immune responses to blood-borne pathogens are initiated [13]. The characteristic splenic architecture encompasses three major compartments: the WP where mostly B and T lymphocytes reside, the RP, populated mainly by F4/80^+^ macrophages, and the MZ, which divides the WP and RP and is populated by MOMA^+^ metallophilic macrophages [13, 14]. The maintenance of this characteristic tissue architecture is important for proper functioning of the spleen and for the generation of immune responses against systemic infections [13, 14]. *Salmonella* has evolved a number of mechanisms by which it evades and suppresses the host’s innate and adaptive immune responses [9, 15–17]. While the innate immune responses arecritical for controlling *Salmonella* at early stages of infection, the initiation of adaptive immune responses and generation of *Salmonella*-specific B and T cell memory is necessary for the host’s long-term protection against subsequent challenges [18–20]. Indeed, *Salmonella*-infected mice exhibit a delayed T cell activation [21, 22]. In addition, memory responses in mice immunized with attenuated *Salmonella* strains develop rather slowly [18, 22], which can be attributed to many mechanisms by which *Salmonella* impairs adaptive immunity.

In this work we show that infection with *Salmonella* leads to the effacement of splenic architecture and causes drastic changes in cell proportions and their in situ distribution. Most notably, RBCs and RP macrophages expand beyond the RP and “take over” WP areas, thus contributing to the re-distribution of lymphocytes in the spleen. In addition, the proportions of B and T lymphocyte populations decrease significantly as infection progresses, likely affecting the initiation of adaptive immune responses. These findings have implications for understanding *Salmonella* pathogenesis and for designing more effective *Salmonella* vaccines.

## Materials and Methods

### Ethics statement

This study was carried out in strict accordance with the recommendations in the Guide for the Care and Use of Laboratory Animals of the National Institutes of Health. The protocol was approved by the Southern Illinois University Institutional Animal Care and Use Committee (Protocol Number: 13–057). Animals were housed in centralized AAALAC-accredited research animal facilities staffed with trained husbandry, technical, and veterinary personnel.

### Mouse and bacterial strains

Six to eight week-old C57BL/6 mice (Jackson Laboratory, Bar Harbor, ME) were used in all experiments. Mice were infected with attenuated *Salmonella* strain χ9088 harboring Asd^+^ plasmid pYA3493 that complemented the Δ*asdA33* mutation in χ9088 [23]. This strain is phenotypically wild-type when grown in the presence of arabinose and becomes attenuated following colonization of host tissues, where free arabinose is not available [23]. *Salmonella* was grown overnight in 5 ml of Luria-Bertani (LB) broth at 37°C. The following day, 100 ml of LB broth was inoculated with an overnight culture (1:100) and grown with aeration at 37°C to an OD_600_ of 0.95. Bacterial cells were pelleted by centrifugation at room temperature (7,000 × g for 15 min), and the pellet was resuspended in 1 ml of buffered saline with gelatin (BSG). To determine the titer of *Salmonella* used for inoculation of mice, dilutions were plated onto LB or MacConkey agar supplemented with 1% lactose.

### Antibodies and reagents

To identify specific cell types, tissues and cell suspensions were stained with combinations of FITC-, PE-, and APC-conjugated monoclonal antibodies. The following antibodies were used to identify specific cell subsets: CD4-PE (clone GK1.5), CD8-PE (clone 53–6.7), biotin and FITC-conjugated F4/80 (clone BM8), Ter119-APC (clone TER-119), CD71-PE (clone R17217) (eBioscience, San Diego, CA), B220-APC (clone RA3-6B2), FITC and PE-conjugated Streptavidin (Biolegend, San Diego, CA), CD169-APC (clone MOMA-1) (AbD Serotec, Raleigh, NC). All antibodies were used at a 1:100 dilution. To highlight the spleen architecture actin-binding phalloidin conjugated to Alexa350 (Invitrogen, Grand Island, NY) was used at a 1:50 dilution.

### Animal infectivity

Mice were infected with either 5x10^5^ (high dose) or 1x10^5^ (low dose) CFUs of *Salmonella* in a 200 μl volume of PBS via a lateral tail vein. CFUs of *Salmonella* in the spleens of infected mice were determined at different times after infection as described previously [24]. Blood samples were collected from uninfected mice (control, day 0) and from infected mice at 3, 6, 9, 14, and 21 days post-infection via a tail vein. Mice were euthanized at 3, 6, 9, 14, and 21 days post-infection and their body and spleen weights were recorded. After weighing, excised spleens were snap-frozen in OCT compound on dry ice and stored at -80°C until sectioning.

### Staining and analysis of frozen spleen sections

For four-color IFM analysis, 5–7 μm-thick spleen tissue sections were fixed in 4% paraformaldehyde for 10 minutes at room temperature, washed three times in PBS then blocked with Blocking Buffer (Thermo Fisher Scientific, Rockford, IL) for 15 minutes. Tissue sections were then stained with panels of monoclonal antibodies for 1 hour, washed in PBS three times and then imaged using a Leica DM4000B fluorescent microscope (Leica Microsystems, Wetzlar, Germany) equipped with Fluotar objectives and QlClick digital CCD camera (QImaging, Surrey, BC, Canada). Monochrome images were acquired with filter sets optimized for 4’, 6-diamidino-2-phenylindole (DAPI), fluorescein isothiocyanate (FITC), tetramethyl rhodamine isothiocyanate (TRITC), and cyanine dye 5 (Cy5) (Chroma, Rockingham, VT), respectively. Acquired monochrome images were imported into Volocity software (PerkinElmer, Waltham, MA) for subsequent color balancing and analysis. For each channel, cell populations were pseudo-colored and fluorescence intensity thresholds were manually set. Cell size and intensity of fluorescence were used to select populations of cells expressing a given cell marker. Numerical data were generated by expressing the number of pixels belonging to a particular cell type as a percentage of the total number of pixels within an image. To identify and quantify CD71^+^, Ter119^+^, and CD71^+^ Ter119^+^ erythroid cells, the selected pixel areas from individual channels (CD71-PE and Ter119-APC) were intersected. The overlapping pixel areas (representing CD71^+^Ter119^+^ cells) were then expressed as percentage of the total pixels per image. Analysis was conducted on 5 representative images from spleen sections of 3 mice for each indicated time point.

### Preparation and imaging of spleen sections by electron microscopy

For transmission electron microscopy (TEM) analysis, spleens of two uninfected (control) mice and two mice infected with 1x10^5^ CFUs of *Salmonella* (day 9 after infection) were cut into 1 mm x 1 mm pieces and rinsed in cold PBS. Tissues were fixed in 2% glutaraldedyde for 2 hours at room temperature and overnight at 4°C. The next day, tissue sections were rinsed four times in Sorensen’s phosphate buffer (0.1 M, pH 7.2) for 30 minutes at room temperature with gentle agitation. The tissues were then post-fixed in 2% osmium tetroxide for 1 hour, rinsed four times with phosphate buffer and 6 times with glass-distilled water for 30 minutes each time. The tissues were then dehydrated in a graded series of ethanol (25%, 50%, 75%, and 100%) for two changes of 20–30 minutes each followed by 1 change of 100% ethanol/propylene oxide (1:1) for 20 minutes and 2 changes of 100% propylene oxide. Following dehydration, the tissues were infiltrated with 2 changes each of Spurr’s embedding resin (EMS, Hatfield, PA) (25%, 50%, 100%) diluted in propylene oxide for 24 hours each. Once in 100% resin, tissues were placed in BEEM capsules (EMS) and polymerized for 24 hours at 60°C. Sixty to 90 nm gold sections were cut using a Leica EM UC7 ultramicrotome (Leica Microsystems, Wetzlar, Germany) fitted with a Diatome diamond knife (Diatome, Hatfield, PA) and transferred to 200 mesh copper grids (EMS). Sections were then post-stained with 2% uranyl acetate for 3 minutes, rinsed for 10 seconds with 0.2 μm filtered distilled water and stained with lead citrate for 30 seconds before drying. Grids were imaged using a Hitachi H-7650 electron microscope. Fifty images were taken from spleens of each mouse, for a total of 400 images, which were analyzed for the presence of macrophages phagocytosing RBCs and lymphocytes.

### Analysis of blood and splenic single cell suspensions with flow cytometry

At day 0, 6, 9, 14, and 21 after infection with 1x10^5^ CFUs of *Salmonella*, spleens and blood samples were collected from 3 mice. A portion of collected blood was used to fill heparinized capillary tubes for determination of PCV. The remainder of the blood sample was used to analyze the profiles of blood RBCs and RBC precursors based on expression of CD71 and Ter119 cell markers. For this, RBCs were pelleted by slow-speed centrifugation for 10 minutes and re-suspended in 100 μl of cold sterile PBS. Cells were then stained for 1 hour with fluorescently-labeled monoclonal antibodies specific for CD71 and Ter119 markers. After staining, cells were washed once in cold PBS then analyzed by flow cytometry. To make splenic single cell suspensions for flow cytometry analysis, spleens were minced, then passed through 70 μm cell strainers (BD Biosciences, San Jose, CA) in cold RPMI-1640. Cells were washed once with cold RPMI-1640 then stained for 1 hour with panels of fluorescently-labeled monoclonal antibodies. Stained cells were analyzed with a BD Accuri C6 flow cytometer (BD Biosciences, San Jose, CA).

### Measurement of Packed Cell Volume (PCV)

At day 0, 9, 14, and 21, blood was collected from *Salmonella*-infected and control mice via a lateral tail vein into two heparinized capillary tubes. The bottom ends of the capillary tubes were sealed with plastacine and the RBCs were pelleted by centrifugation. PCV (%) was determined by dividing the packed RBC volume by the total blood volume loaded in the capillary tube.

### Statistical analysis

Data were analyzed by analysis of variance (ANOVA) procedures using JMP software (SAS Institute, Cary, NC). Group means were separated by Tukey's multiple-comparison procedure and declared significantly different at p<0.05. Data are expressed as the mean ± standard deviation of the mean.

## Results

### Infection with *Salmonella* causes anemia and alters the proportions of peripheral RBC populations

**Fig 1:**
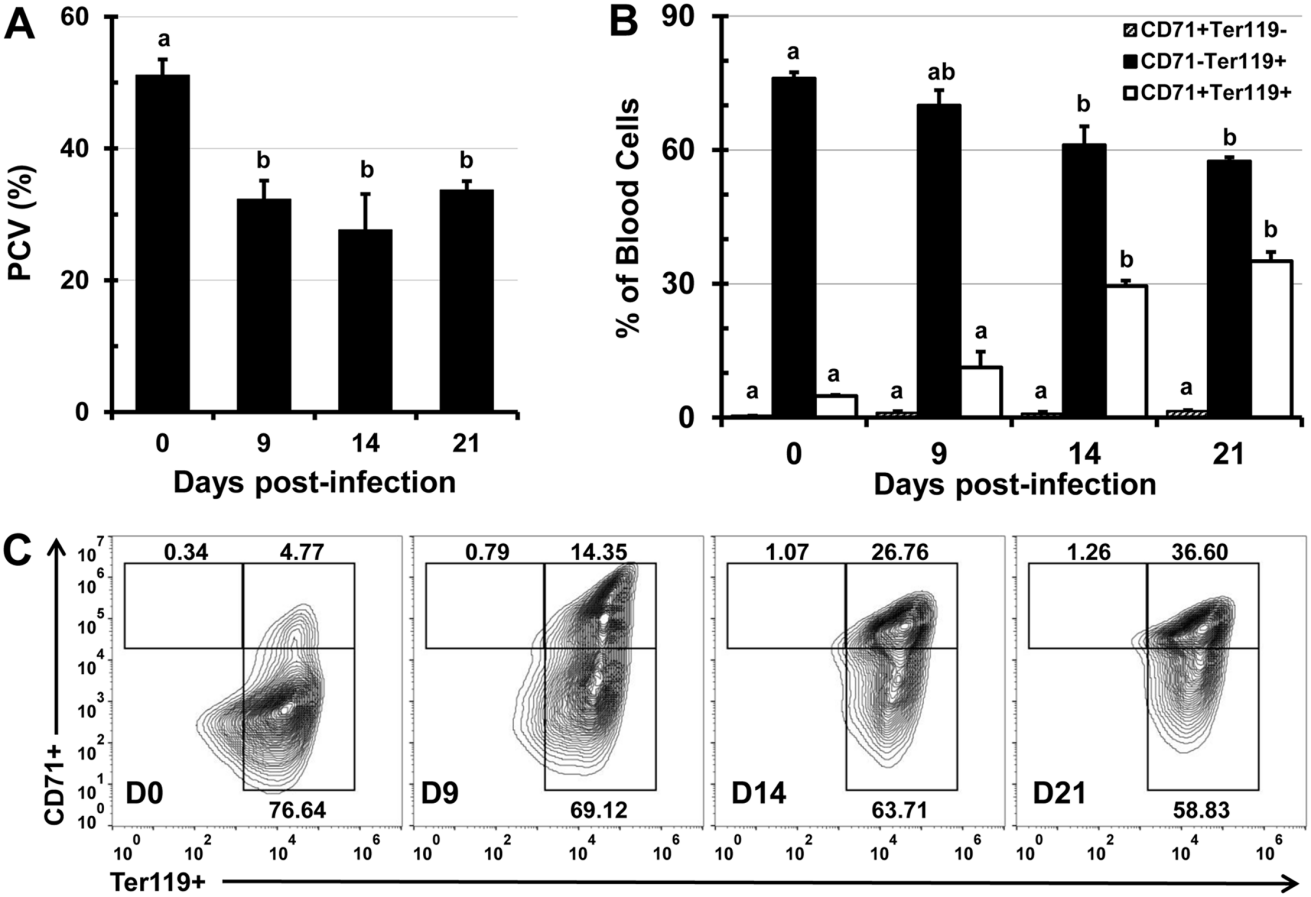
Infection with *Salmonella* causes anemia and alters proportions of erythroid cell populations in peripheral blood. C57BL/6 mice were infected i.v. with 1x10^5^ CFUs of *Salmonella* and at day 0 (uninfected control), 9, 14, and 21, blood samples were collected. (A) PCV (%) was calculated by dividing packed cell volume by total blood volume. (B) Blood samples were stained with fluorescent antibodies specific for erythroid cell markers CD71 and Ter119, then analyzed by flow cytometry. Proportions of CD71^+^ Ter119^-^, CD71^+^ Ter119^+^, and CD71^-^ Ter119^+^ reticulocytes are shown as means ± standard deviation (n = 3 mice per time point). (C) Representative flow cytometry contour plots of blood samples taken at day 0, 9, 14, and 21 post-infection. Group means that do not share superscripts are significantly different from each other (p< 0.05).

Anemia of inflammation (AI) is characterized by low serum iron concentration and inadequate RBC production which is reflected in decreased PCV and an increased proportion of immature RBCs (CD71^+^ Ter119^+^) in peripheral blood. Acute sepsis causes AI that is refractory to erythropoiesis-stimulating agents, thus administration of bacterial LPS abolishes iron use and erythropoiesis, even when LPS is administered with EPO analogs [25]. Infection with *Salmonella* was reported to significantly elevate systemic EPO concentration, without substantially altering the proportions of Ter119^+^ erythroid cells in peripheral blood [5]. Since the Ter119 marker is expressed in both mature (CD71^-^ Ter119^+^) and immature (CD71^+^ Ter119^+^) erythroid cells, it is not possible to determine whether *Salmonella* alters the proportions of erythroid subsets in peripheral blood by analyzing Ter119 expression alone. To address this question we measured the volume (%) of RBCs in whole blood (PCV) and analyzed the erythroid populations in peripheral blood based on the expression of CD71 and Ter119 markers. Within the first week of infection, mice became anemic and the PCV decreased by 37% and 46% by weeks 1 and 2 after infection, respectively (Fig 1A). At three weeks after infection, as *Salmonella* titers in the tissue declined (S1 Fig), the PCV started to increase, but still remained significantly lower compared to the PCV of uninfected control mice (Fig 1A). In addition, kidneys of infected mice had a pale nude color, in comparison to the reddish colored kidneys of control mice (not shown), indicating tissue hypoxia. The decreased PCV was accompanied with significant changes in the proportions of Ter119^+^ peripheral erythroid cells. Most notably, the proportions of CD71^+^ Ter119^+^ immature erythroid cells increased from an average of 5% to 35% (Fig 1B and 1C), while proportions of mature CD71^-^ Ter119^+^ RBCs decreased by 20% during 21 days of infection (Fig 1B and 1C). The proportions of CD71^+^ Ter119^-^ cells in peripheral blood were low and did not substantially change (Fig 1B and 1C).

### Infection with *Salmonella* causes extramedullary erythropoiesis, splenomegaly, and effacement of splenic microarchitecture

**Fig 2:**
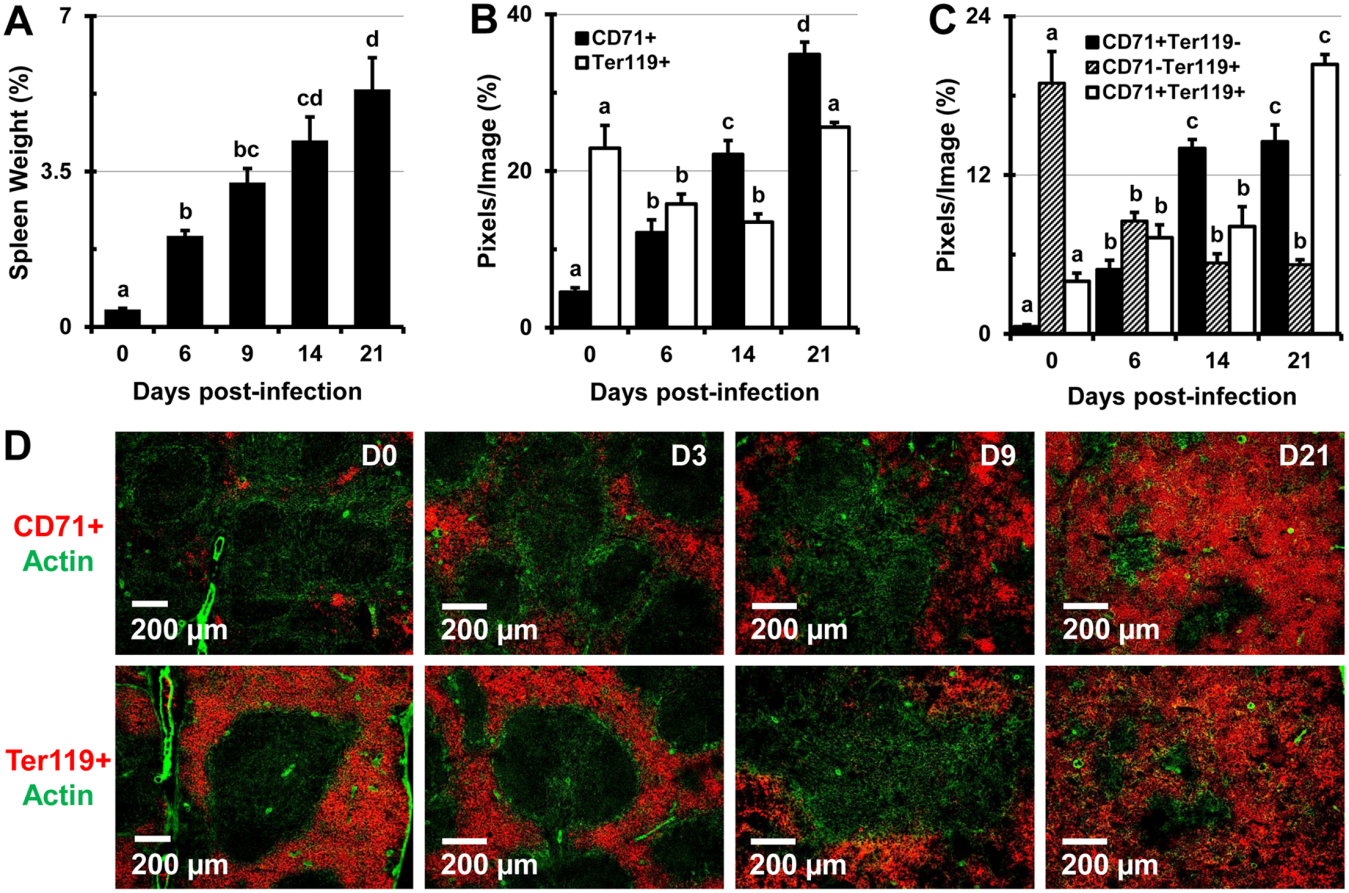
Infection with *Salmonella* causes marked increase in spleen size and alters the proportions of erythroid cells in the spleens of C57BL/6 mice. (A) Spleens of uninfected control mice (day 0) and mice infected with 1x10^5^ CFUs of *Salmonella* were excised and weighed at 0, 6, 9, 14, and 21 days post-infection. Spleen weights are shown as a percentage of body weight. Data are expressed as the mean ± standard deviation (n = 3 mice per time point). (B-D) Spleen tissue sections were stained with panels of 3 fluorescently tagged monoclonal antibodies and images were analyzed using Volocity software. The percentage of image area containing cells expressing erythroid cell markers was calculated from 5 images per mouse, per cell marker (n = 3 mice per time point). (B) Proportions of CD71^+^, Ter119^+^, and (C) CD71^+^ Ter119^-^, CD71^-^ Ter119^+^, CD71^+^ Ter119^+^ erythroid cell populations are expressed as the mean ± standard deviation (n = 3 mice per time point). Group means that do not share a superscript are significantly different from each other (p< 0.05). (D) Representative images showing CD71^+^ (top row, red) and Ter119^+^ (bottom row, red) erythroid cells in spleen sections at day 0 (control), 3, 9, and 21 post-infection. Actin staining with phalloidin-Alexa350 (green) highlights the tissue architecture.

In agreement with a previous a report [5], we found that extramedullary erythropoiesis is one of the main causes of splenomegaly. After one week of infection with 1x10^5^ CFUs of *Salmonella*, the spleens of mice increased in size by about 5-fold, and by three weeks after infection the spleen increased in size by over 10-fold, at which time it comprised about 5.3% of total body weight (Fig 2A). At later stages of infection some mice exhibited pathological splenic rupture observed during spleen excision (S2 Fig). In the spleens of mice infected with 5x10^5^ (high dose, not shown) and 1x10^5^ (low dose) CFUs of *Salmonella* there was a significant increase in the proportions of CD71^+^, and a decrease in proportions of Ter119^+^ erythroid cells (Fig 2B). Two color colocalization analysis of tissue sections revealed that the proportions of dual positive (CD71^+^ Ter119^+^) immature RBCs, as well as earlier RBC precursors (CD71^+^ Ter119^-^) increased as infection progressed (Fig 2C). However, the mature RBC population (CD71^-^ Ter119^+^) decreased by almost 75% in mice infected with 1x10^5^ CFUs of *Salmonella* (Fig 2C), and by about 80% in mice infected with 5x10^5^ CFU of *Salmonella* (not shown). In uninfected mice (control) CD71^+^ erythroid progenitors were mainly located in erythroid niches within the RP, in close proximity to the MZ (Fig 2D), however as infection progressed they expanded not only in the RP, but also in the WP, making it impossible to delineate these regions in spleen tissue sections (Fig 2D).

### *Salmonella* infection leads to drastic alterations in the proportion and distribution of the F4/80^+^ and MOMA^+^ macrophages in the spleen

**Fig 3:**
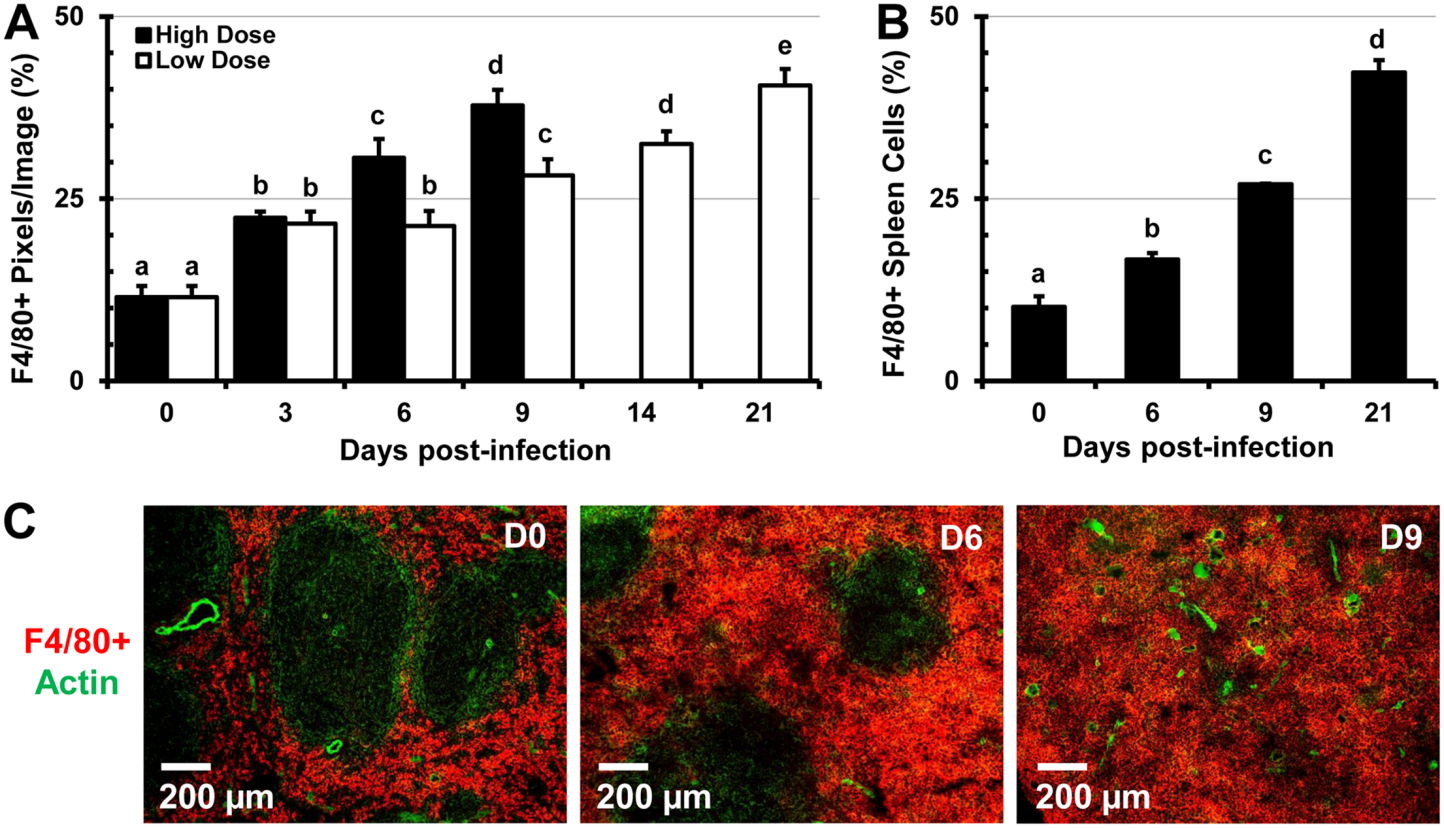
Increased proportions of F4/80^+^ splenic RP macrophages during *Salmonella* infection. C57BL/6 mice were infected with 5x10^5^ CFU (high dose) or 1x10^5^ CFUs (low dose) of *Salmonella* and were euthanized 3, 6, 9, 14, and 21 days post-infection along with uninfected controls (day 0). (A) Images were analyzed using Volocity software and proportions of F4/80^+^ cells are expressed as the percentage of pixels (image area) per total image area from 5 images per mouse (n = 3 mice per time point). (B) Single cell suspensions from spleens of C57BL/6 mice infected with 1x10^5^ CFUs of *Salmonella* and uninfected controls (day 0) were stained with antibodies specific for the F4/80 cell marker and analyzed by flow cytometry. Data are expressed as the mean ± standard deviation (n = 3 mice per time point). Group means that do not share a superscript are significantly different from each other (p< 0.05). (C) In situ changes in the distribution of F4/80^+^ macrophages (red) at day 0 (control), 6, and 9 post-infection with 1x10^5^ CFUs of *Salmonella*. Actin staining with phalloidin-Alexa350 (green) highlights the tissue architecture of the spleen.

**Fig 4:**
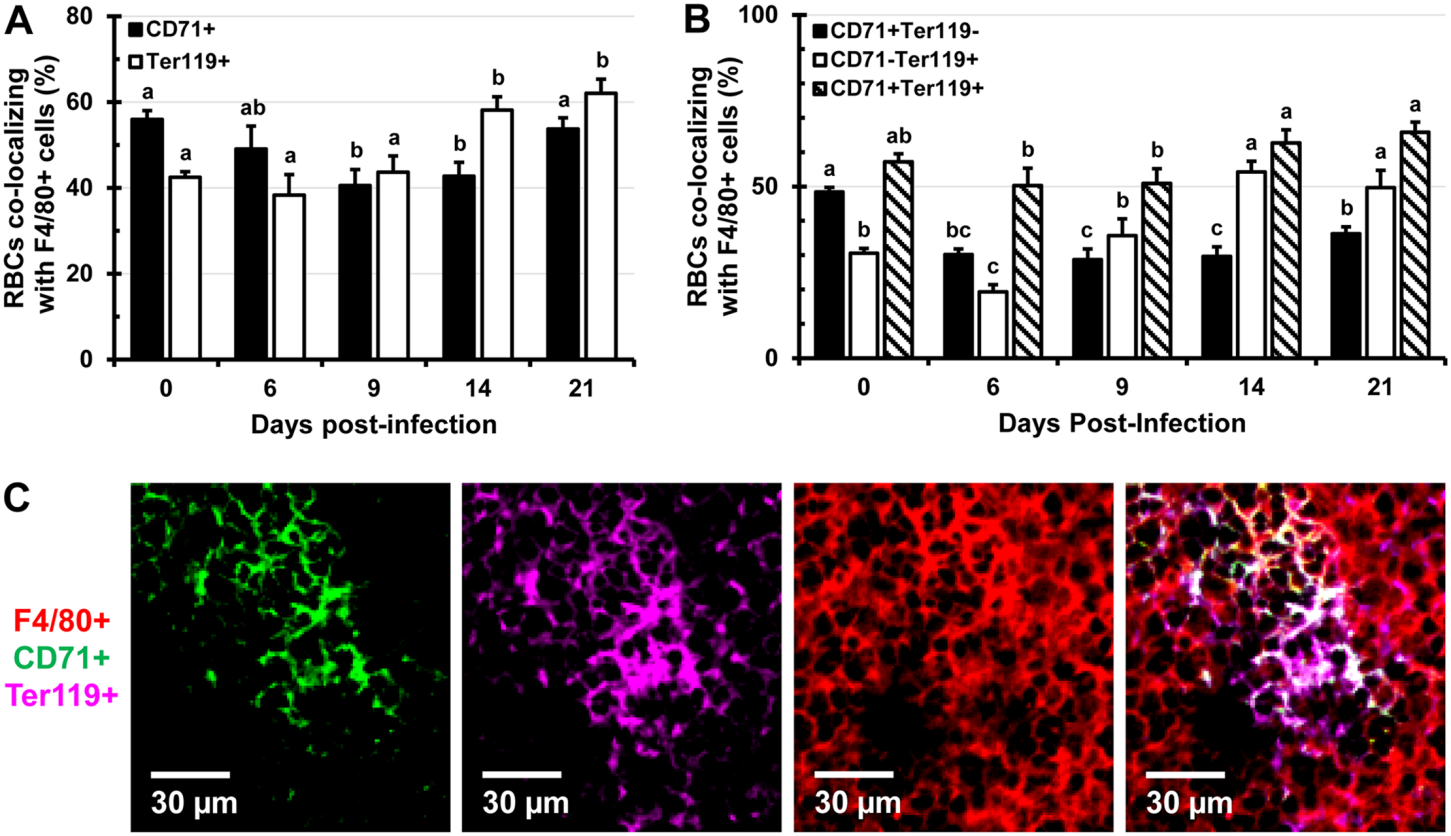
Colocalization of F4/80^+^ macrophages with RBCs and their immature precursors during infection with *Salmonella*. C57BL/6 mice were infected i.v. with 1x10^5^ CFUs of *Salmonella* and spleen tissue sections were stained with antibodies specific for CD71, Ter119, and F4/80 cell markers. Two (A) and three color (B) colocalization analysis was performed using Volocity software. Data are expressed as the percentage of pixels positive for CD71 or Ter119 colocalizing with pixels positive for F4/80 (A) or as the percentage of pixels positive for CD71 and/or Ter119 colocalizing with pixels positive for the F4/80 cell marker (B). Data are expressed as the mean ± standard deviation of 5 images analyzed per mouse (n = 3 mice per time point). Group means that do not share a superscript are significantly different from each other (p< 0.05). (C) A representative 3 color image showing colocalization of CD71^+^ (green, panel 1) and Ter119^+^ (purple, panel 2) cells with F4/80^+^ (red, panel 3) macrophages. Panel 4 shows overlap of panels 1–3.

**Fig 5:**
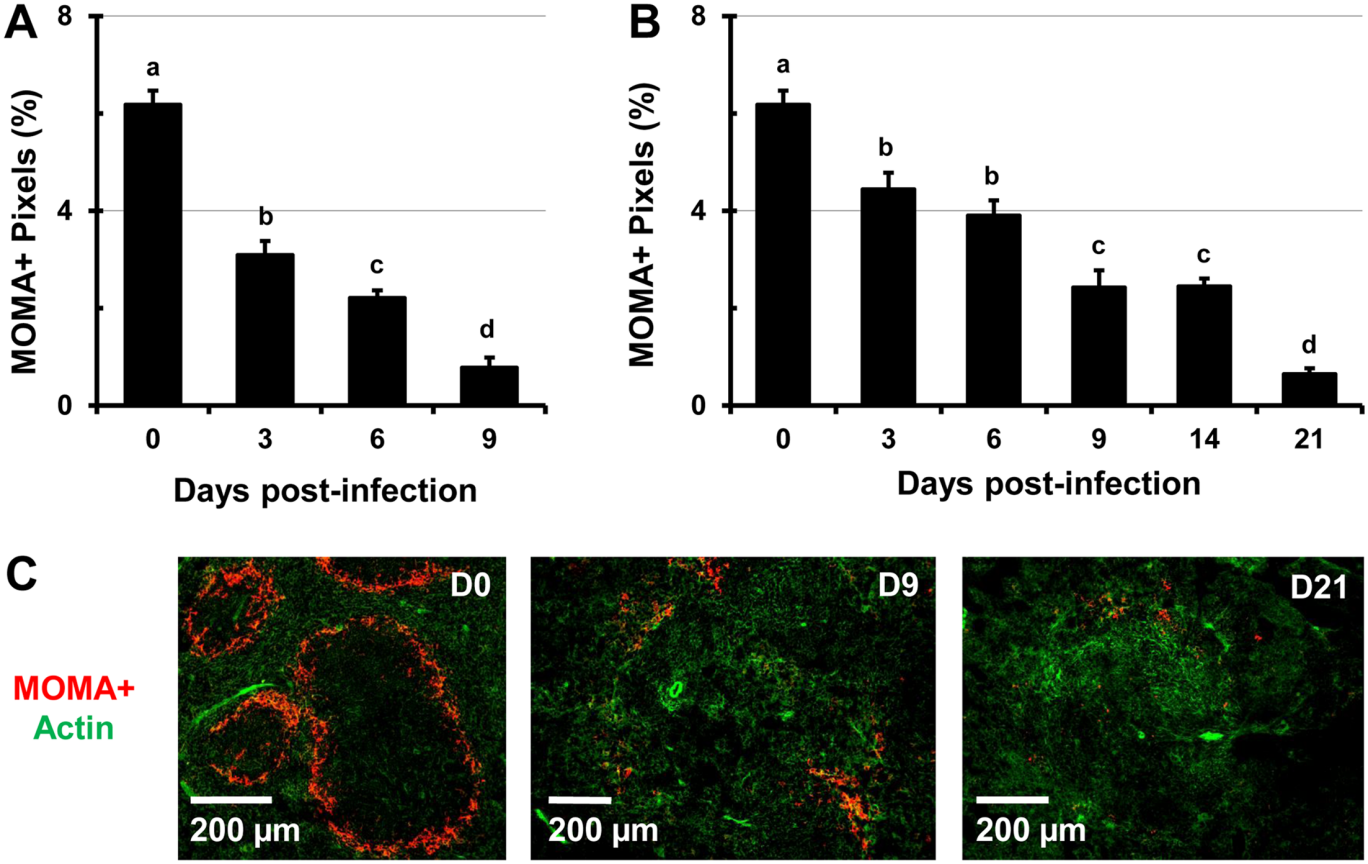
Decreased proportions of MOMA^+^ MZ macrophages during infection with *Salmonella*. C57BL/6 mice were infected i.v. with (A) 5x10^5^ CFUs (high dose) or (B) 1x10^5^ CFUs (low dose) of *Salmonella* and spleen sections were stained with MOMA antibodies. Images were analyzed using Volocity software and data are expressed as the percentage of pixels positive for the MOMA cell marker per total image area. Data is expressed as the mean ± standard deviation. Means within a group that do not share superscripts are significantly different from each other (p< 0.05). (C) Representative images showing the changes in the distribution of MOMA^+^ macrophages (red) at day 0 (control), 6, and 9 post-infection in spleens of mice infected with 5x10^5^ CFUs of *Salmonella*. Actin staining with phalloidin-Alexa350 (green) highlights the tissue architecture of the spleen.

The spleen contains heterogeneous populations of macrophages that occupy discrete anatomical regions [26]. The MZ of the spleen is populated by marginal-zone MOMA^+^ macrophages which do not express the F4/80 marker and are involved in the capture of blood-borne pathogens [13, 27]. In the RP of the spleen, F4/80^+^ macrophages predominate and play an essential role in the clearance of senescent erythrocytes and iron recycling [26, 27]. During the course of infection with high (9 days) and low doses (21 days) of *Salmonella*, the proportion of F4/80^+^ macrophages increased by over 3-fold (Fig 3A and 3B). In spleens of control mice, macrophages are smaller and more compact, while in spleens of *Salmonella*-infected mice, macrophages increasingly predominate and are greatly enlarged (S3 Fig). Mirroring the expansion of erythroid cells, F4/80^+^ macrophages expanded beyond the RP and encroached into the WP area of the spleen (Fig 3C and S4 Fig). Two color IFM analysis revealed that over time the colocalization of F4/80^+^ macrophages with CD71^+^ RBC progenitors decreased up to day 14 of infection, while colocalization with Ter119^+^ erythroid cells increased up to day 21 (Fig 4A). Examining spleen sections stained for F4/80, CD71, and Ter119 (S4 Fig) we found that over time CD71^+^ Ter119^+^ and CD71^-^ Ter119^+^ erythroid cells increasingly colocalized with F4/80^+^ macrophages, which can be seen actively phagocytosing RBCs (S3 and S5 Figs). In contrast, colocalization of earlier erythroid progenitors (CD71^+^ Ter119^-^) with F4/80^+^ macrophages decreased over the course of the study (Fig 4B and 4C). As infection with *Salmonella* progressed, the proportions of MOMA^+^ MZ macrophages declined significantly and in a dose-dependent manner (Fig 5A and 5B). In spleen sections of uninfected mice (controls), MOMA^+^ macrophages form prominent “rings” that separate the WP from the RP (Fig 5C), which disappear in spleens of *Salmonella*-infected mice due to decrease of MOMA^+^ cells (Fig 5C).

### Infection with *Salmonella* leads to a decrease in proportions of B and T lymphocytes

**Fig 6:**
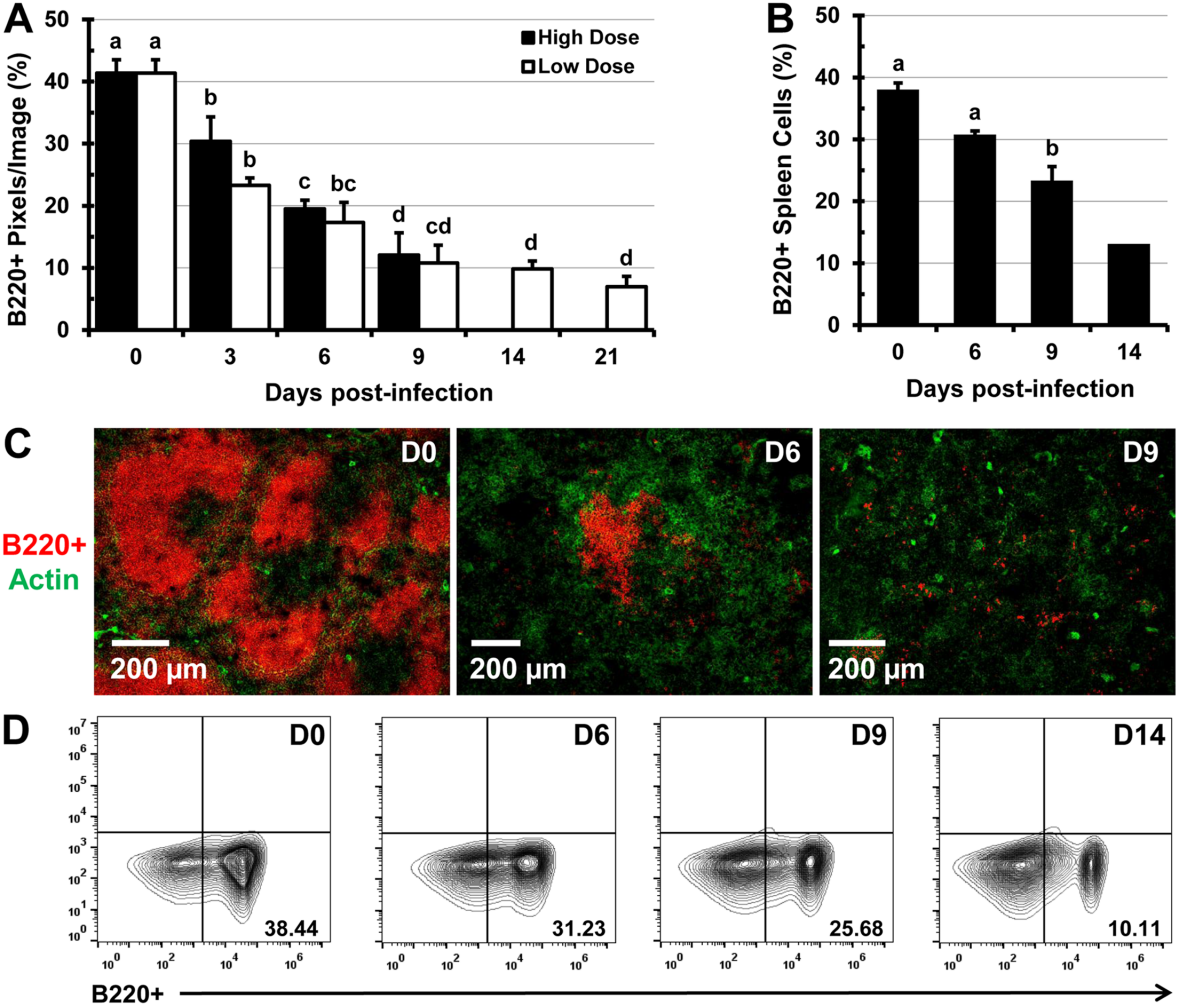
Infection with *Salmonella* leads to the destruction of B cell follicles in the spleen and diminishes the proportions of B220^+^ lymphocytes. C57BL/6 mice were infected i.v. with 5x10^5^ CFUs (high dose) or 1x10^5^ CFUs (low dose) of *Salmonella* and at 0 (uninfected control), 3, 6, 9, 14, and 21 days post-infection, spleen sections were stained with monoclonal antibodies specific for the B220 cell marker. (A) Five images per mouse spleen (n = 3 mice per time point) were analyzed using Volocity software and the image area containing B220^+^ lymphocytes (red) was expressed as a percent of the total image area. (B) Spleen single-cell suspensions were stained with antibodies specific for the B220 cell marker and analyzed by flow cytometry. Data are expressed as the mean ± standard deviation (n = 3 mice per time point. The mean value for day 14 is averaged from 2 mice. Group means that do not share a superscript are significantly different from each other (p< 0.05). (C) Representative images of spleen tissue sections from uninfected mice (day 0) or mice infected with 5x10^5^ CFUs of *Salmonella* (B220^+^ B cells, red). Actin staining with phalloidin-Alexa350 (green) highlights the tissue architecture. (D) Representative flow cytometry contour plots showing proportions of B220^+^ splenocytes at day 0, 6, 9, and 14 post-infection.

**Fig 7:**
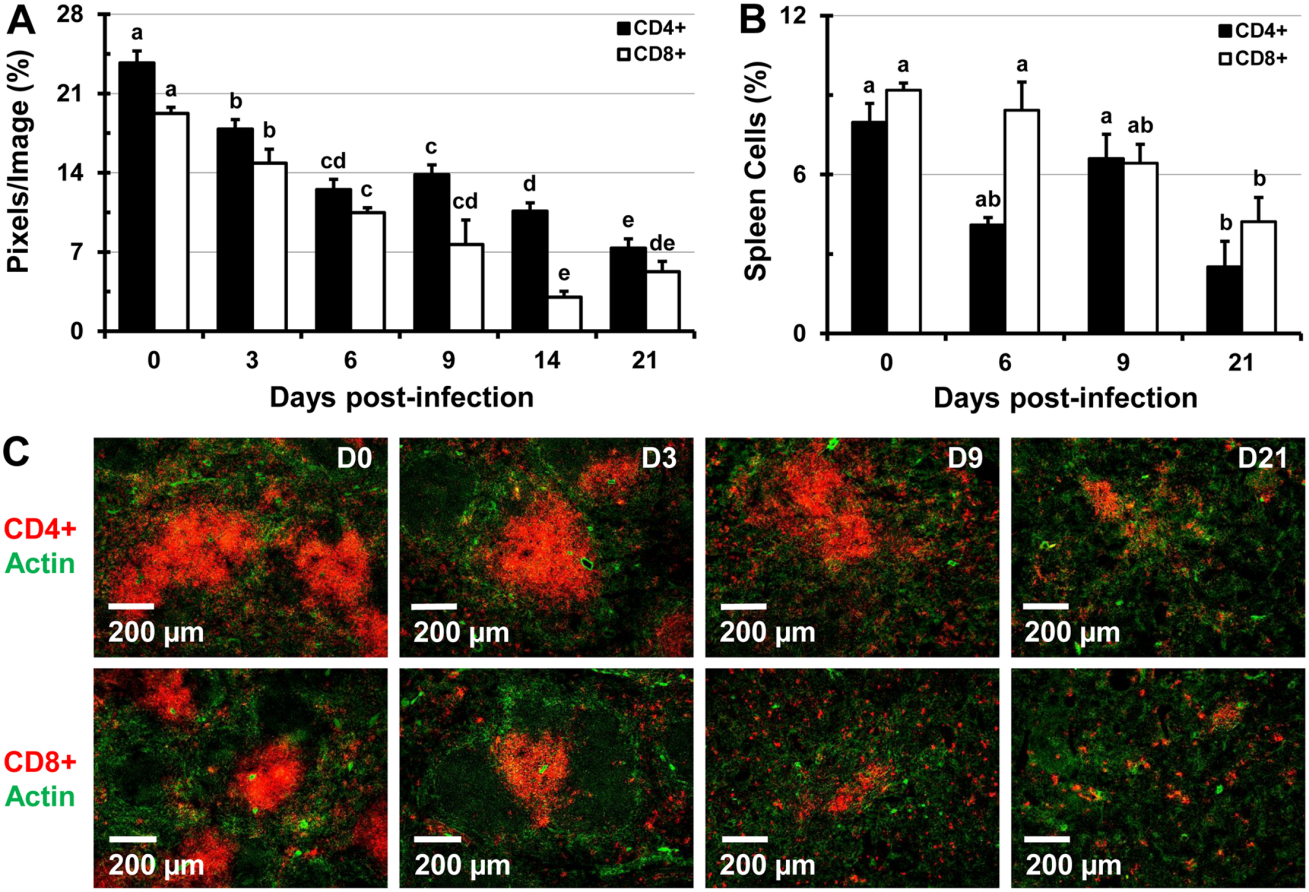
Infection with *Salmonella* leads to decreased proportions of CD4^+^ and CD8^+^ T lymphocytes in the spleen. Spleens of C57BL/6 mice infected with 1x10^5^ CFUs of *Salmonella* were stained with antibodies specific for CD4 and CD8 cell markers. (A) Images were analyzed using Volocity software and the image area (pixels) containing CD4^+^ or CD8^+^ T lymphocytes were expressed as a percentage of the total area from 5 images per mouse spleen (n = 3 mice per time point). (B) Proportions of CD4^+^ and CD8^+^ splenocytes in cell suspensions of C57BL/6 mice infected with 1x10^5^ CFUs of *Salmonella* at day 0 (control), 6, 9, and 21 days post-infection analyzed by flow cytometry. Data are expressed as the mean ± standard deviation (n = 3 mice per time point). Group means that do not share a superscript are significantly different from each other (p< 0.05). (C) Representative images of spleens from control mice and mice infected with 1x10^5^ CFUs of *Salmonella* showing changes in the proportions of CD4^+^ (top row, red) or CD8^+^ cells (bottom row, red). Actin staining with phalloidin-Alexa350 (green) highlights the tissue architecture.

Analysis of fluorescently-labeled spleen sections using Volocity revealed that during the course of infection with *Salmonella* (low and high dose) the B and T lymphocyte proportions decline. A significant decrease in the proportion of B220^+^ B lymphocytes was seen by day 3 of infection, a trend that continued up to day 21 of infection (Fig 6A and 6C). This finding was also confirmed by flow cytometry analysis of splenic single cell suspensions (Fig 6B and 6D). In addition, infection with *Salmonella* led to a significant decline in the proportions of CD4^+^ and CD8^+^ T lymphocytes in stained tissue sections of spleens analyzed by IFM (Fig 7A and 7C) and in splenic cell suspensions analyzed by flow cytometry (Fig 7B). The dramatic disappearance of characteristic B and T cell follicles was accompanied with re-distribution of B and T cells within the spleen (Figs 6C and 7C, S6 Fig). Flow cytometry analysis revealed that while total numbers of cells per spleen, RBCs, and F4/80^+^ macrophages increase (day 0 to 14 of infection), the numbers of T and B lymphocytes increase up to day 9, then decline up to day 14 of infection (not shown).

### B and T lymphocytes increasingly colocalize with F4/80^+^ macrophages as infection progresses

**Fig 8:**
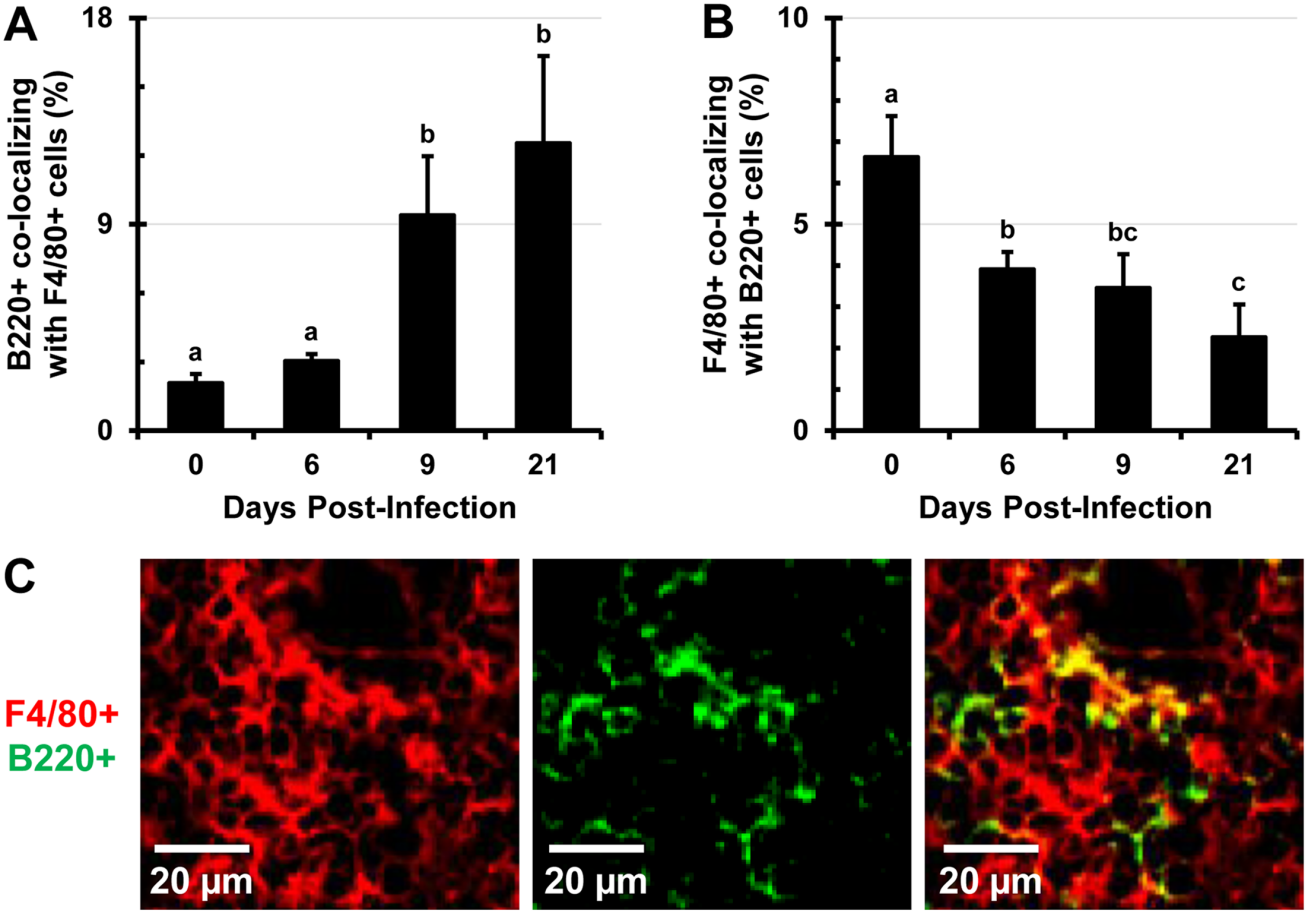
Colocalization of B220^+^ lymphocytes with F4/80^+^ macrophages increases as infection with *Salmonella* persists. Mice were i.v. infected with 1x10^5^ CFUs of *Salmonella* and at day 0 (uninfected control), 6, 9, and 21, spleen tissue sections were stained with antibodies specific for the B220 lymphocyte marker and F4/80 macrophage marker. Stained sections were analyzed with Volocity software and data are expressed as the percentage of pixels positive for the B220 marker colocalizing with the pixels positive for the F4/80 cell marker (A). (B) Percentage of F4/80^+^ pixels colocalizing with B220^+^ pixels. (C) A representative image of in situ colocalization of B220^+^ lymphocytes (green) with F4/80^+^ macrophages (red). Five images from each individual mouse were analyzed (n = 3 mice per time point). Data are expressed as the mean ± standard deviation. Group means that do not share a subscript are significantly different (p<0.05).

**Fig 9:**
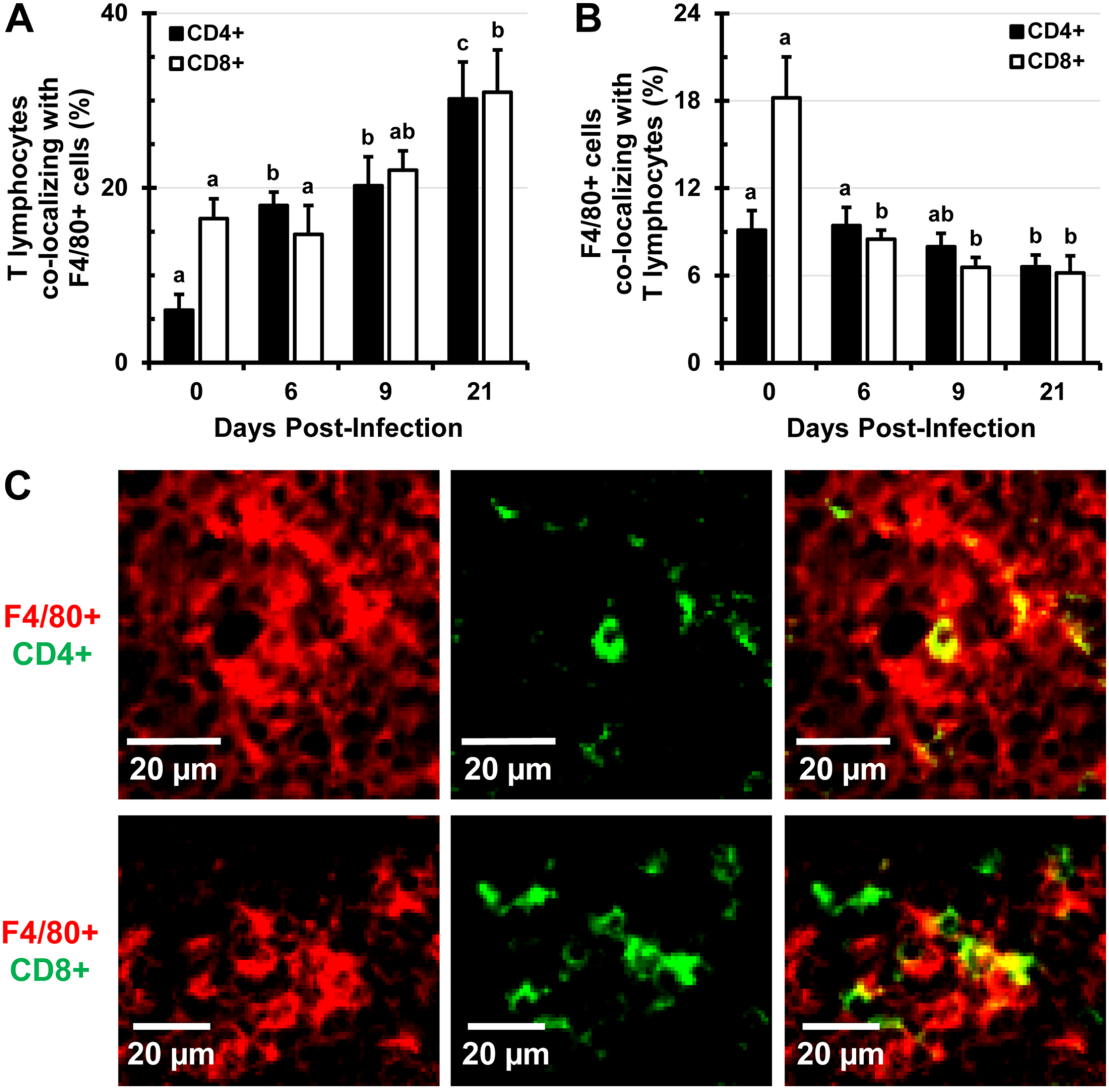
Colocalization of CD4^+^ and CD8^+^ lymphocytes with F4/80^+^ macrophages increases as infection with *Salmonella* persists. Mice were infected i.v. with 1x10^5^ CFUs of *Salmonella* and at day 0 (uninfected control), 6, 9, and 21, spleen tissue sections were stained with antibodies specific for CD4 and CD8 lymphocyte markers, as well as the F4/80 macrophage marker. Stained sections were analyzed with Volocity software and data are expressed as the percentage of pixels positive for the CD4 or CD8 marker colocalizing with pixels positive for the F4/80 cell marker (A). (B) Percentage of F4/80^+^ pixels colocalizing with CD4^+^ and CD8^+^ pixels. (C) A representative image of in situ colocalization of CD4^+^ lymphocytes (top panel, green) with F4/80^+^ macrophages (top panel, red) and CD8^+^ lymphocytes (bottom panel, green) with F4/80^+^ macrophages (bottom panel, red). Five images from individual mice were analyzed (n = 3 mice per time point). Data are expressed as the mean ± standard deviation. Group means that do not share a subscript are significantly different (p< 0.05).

**Fig 10:**
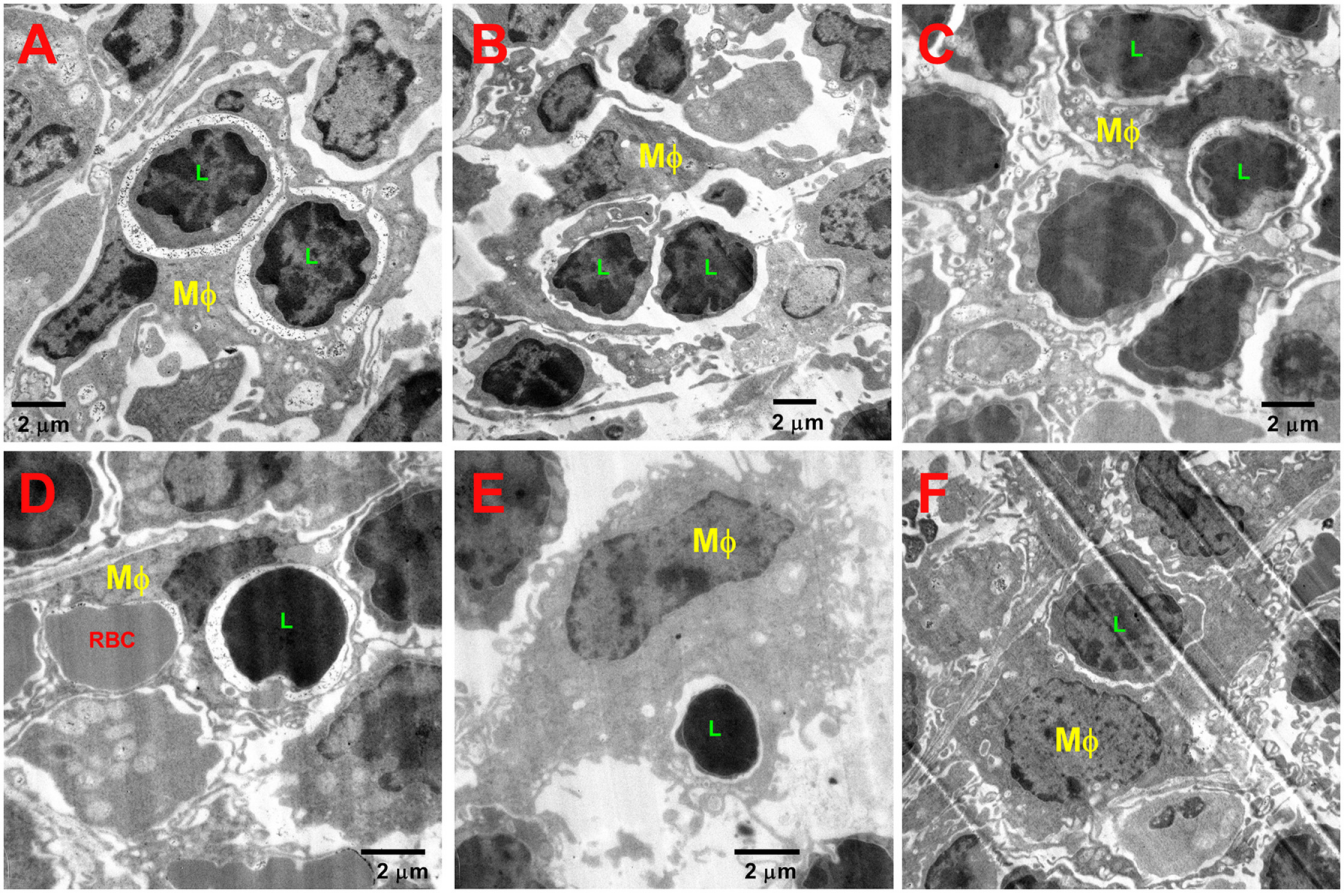
TEM images of spleen sections of *Salmonella*-infected mice. (A) A macrophage (Mϕ) with two phagocytosed lymphocytes (L). (B) A macrophage wrapping itself around two lymphocytes. (C) A macrophage with two phagocytosed lymphocytes. (D) A macrophage with a phagocytosed lymphocyte and RBC. (E) A lymphocyte within a macrophage’s cytoplasm. (F) A macrophage that has phagocytosed a lymphocyte.

The expansion of F4/80^+^ macrophages and erythroid cells beyond the RP and into the WP is accompanied with alterations in the distribution and proportions of B and T lymphocytes. There is an increase of in situ colocalization of B lymphocytes with F4/80^+^ macrophages from about 2.5% in uninfected controls to about 12% at day 21 of infection (Fig 8A and 8C, S7 Fig top row). In contrast to this, the proportion of expanding F4/80^+^ macrophages harboring B220^+^ B lymphocytes in the spleen declines significantly as infection persists and the proportion of B lymphocytes dwindle (Fig 8B). Similarly, colocalization of CD4^+^ and CD8^+^ lymphocytes with F4/80^+^ macrophages increases over the course of infection (Fig 9A and 9C, S7 Fig middle and bottom rows), while the proportions of F4/80^+^ macrophages colocalizing with CD4^+^ or CD8^+^ lymphocytes decrease, as proportions of these cells decline (Fig 9B). This finding was also confirmed by confocal microscopy (not shown). TEM imaging revealed that in spleens of *Salmonella*-infected mice, macrophages can be seen phagocytosing lymphocytes very frequently (Fig 10). In spleens of uninfected (control) mice only one instance of a lymphocyte phagocytosis by a macrophage was observed from about 100 images analyzed (not shown). Enlarged macrophages often appear to contain more than one nucleus, resemble giant macrophages, and are observed surrounding lymphocytes and RBCs (S8 Fig).

## Discussion

Clearance of *Salmonella* depends on coordinated actions of innate and adaptive immune responses of the infected hosts [28]. However, like many other bacterial pathogens, *Salmonella* has evolved a number of mechanisms which enable it to evade host immune responses, persist, and cause disease. In addition to a number of disease symptoms, *Salmonella* infection causes extramedullary erythropoiesis and splenomegaly [4, 5]. The pathology of splenomegaly involves a complex interplay of host-pathogen interactions, with LPS and EPO playing important roles [5]. The increased EPO production, induced by LPS signaling via Myd88/TRIF was shown to cause an increase in splenic leukocyte and erythroid cell numbers, without substantially altering proportions of blood erythroid cell concentrations [5]. Bacterial LPS alone was also shown to induce NO production, cellular apoptosis, and renal hypoxia [7]. Renal tubular interstitial cells sense the low tissue oxygen levels and secrete EPO in order to increase the generation of new RBCs, thus enhancing tissue oxygenation [8, 29]. Therefore, administration of EPO reduces LPS-induced tissue hypoxia, NO production, and apoptosis [7]. On the other hand, LPS suppresses the activity of erythropoiesis-stimulating agents (EPO analogs), leading to decreased systemic hemoglobin concentrations and RBC proportions [25]. Anemia caused by infection or inflammation is characterized by EPO hyporesponsiveness, reduced proliferation of erythroid precursors, and decreased RBC survival. A major contributor of anemia induced by infection is hepcidin, a hormone that inhibits the release of stored cellular iron and iron transfer from enterocytes to systemic circulation. In addition, both iron deficiency and sepsis can enhance eryptosis, exacerbating anemia, if not compensated by erythropoiesis [30]. Iron is essential for the survival and pathogenesis of bacteria, including *Salmonella*, thus iron withdrawal from circulation mediated by hepcidin is a host-protective mechanism. Infection with *Salmonella* also causes anemia [4] and reticulocytosis, characterized by a significant decrease in blood PCV (hematocrit), increased proportions of CD71^+^ Ter119^+^ immature RBCs, and decreased proportions of CD71^-^ Ter119^+^ mature RBCs in peripheral blood. The decline in systemic CD71^-^ Ter119^+^ mature RBC proportions may be partly due to *Salmonella*-induced oxidative stress coupled with elevated EPO, likely leading to the formation of RBCs which are sensitive to eryptotic stimuli. Increased proportions of systemic immature RBCs and decreased PCV indicate enhanced, however ineffective, erythropoiesis. The increased proportions of CD71^+^ Ter119^+^ and CD71^+^ Ter119^-^ immature RBC precursors in the spleens of *Salmonella*-infected mice indicates active extramedullary erythropoiesis. As reported previously [5], the expansion of RBC precursors is a major cause of the observed splenomegaly. In addition, hemophagocytic F4/80^+^ RP macrophages contribute considerably to splenomegaly, as this subset of macrophages significantly increases during infection with *Salmonella*. In addition to removing blood-borne pathogens, one of the main functions of F4/80^+^ RP macrophages is to phagocytose aged or damaged RBCs, degrade their hemoglobin and extract the iron [27, 31], thus they highly express the genes involved in iron regulation [32]. Loss of F4/80^+^ RP macrophages in Spi-C^-/-^ mice leads to inefficient RBC phagocytosis in the spleen, iron overload, and splenomegaly [32]. Type I interferon-dependent killing of splenic F4/80^+^ macrophages by *Salmonella* [33] might also increase the levels of free heme, which induces the expression of Spi-C in monocytes, leading to the generation of new RP and bone marrow macrophages [34]. Thus, in spite of *Samonella*-induced death of F4/80^+^ macrophages, their proportions increase, indicating that F4/80^+^ macrophages are replenished either by blood monocytes, or possibly by local tissue macrophage proliferation [35]. EPO (which is elevated during infection with *Salmonella*) was shown to increase the numbers of splenic macrophages, upregulate the expression of F4/80, CD11b, and CD80 cell markers, and enhance their pro-inflammatory and phagocytic activity [36]. Thus, EPO likely contributes to the expansion of F4/80^+^ macrophages in spleens of *Salmonella*-infected mice which increasingly colocalize with erythroid cells, indicating their active phagocytosis by F4/80^+^ macrophages. The higher proportions of F4/80^+^ macrophages allows for the processing of heme and iron sequestration, as excessive accumulation of intracellular heme can be toxic to macrophages [37]. As infection with *Salmonella* persists, erythroid cells and F4/80^+^ macrophages expand beyond the RP and “take over” the WP, thus drastically altering the characteristic splenic architecture comprised of the MZ, RP, and WP. Moreover, a marked decrease in proportions of MZ MOMA^+^ macrophages further disrupted the tissue microarchitecture. Remodeling of the splenic architecture, loss of T and B lymphocyte compartmentalization, and depletion of MZ macrophages is also observed during an infection with murine cytomegalovirus (MCMV) [38]. In addition, *Leishmania donovani* causes depletion of MZ macrophages in a TNF-α-dependent manner causing extensive tissue remodeling resulting in impaired lymphocyte traffic into the white pulp during chronic leishmaniasis [39]. Infection with *Salmonella* stimulates endogenous production of TNF-α and other toxic mediators in the host. Activated macrophages produce a significant amount of TNF-α, but they also contribute to the release of oxygen radicals and NO, which are toxic to pathogens, as well as to the host cells [34, 40, 41]. Thus, the excessive release of bactericidal toxic mediators in vivo can lead to localized tissue destruction, which might be a reason for the depletion of MOMA^+^ macrophages, loss of B and T lymphocyte compartmentalization, and the destruction of splenic tissue architecture. In addition, WP B and T cell follicles shrink and eventually disappear, as proportions of both B and T lymphocytes decline due to a persistent infection. The splenic tissue architecture is important not only for the capture of blood-borne pathogens, but also for facilitating the interactions between lymphocytes and antigen-presenting cells, which are essential for the establishment of protective immunity against *Salmonella* [6, 42–44]. *Salmonella* can also hinder the activation of the adaptive immune response [22, 45] by inhibiting DC functions and the activation of T cells [16], by infecting B cells and inhibiting their proliferation [46, 47], and by reducing the ability of T cells to proliferate and produce cytokines [9, 48]. Increased phagocytosis of B and T lymphocytes by expanding F4/80^+^ RP macrophages coupled with the inhibition of lymphocyte proliferation may account for declining lymphocyte numbers during *Salmonella* infection. As infection persists, proportions of B and T lymphocytes that harbor *Salmonella* increase (unpublished observations), thus phagocytosis of lymphocytes by RP macrophages may be a means of controlling the dissemination and persistence of *Salmonella*. Lymphocytes may indeed provide a survival niche for *Salmonella* during chronic infection, as it was shown that *Salmonella* preferentially resides and replicates in hemophagocytic macrophages that have phagocytosed live cells, but not in macrophages that phagocytosed dead cells or inert particles [49, 50].

*Salmonella* strain χ9088 used in these studies is a vaccine strain engineered to be administered per orally and as such, it induces protective immunity to a challenge with wild-type *Salmonella* [23]. Since this strain is highly attenuated, mice survived the i.v. administration of a relatively large dose (1x10^5^ CFUs) for over 3 weeks, which allowed us to examine changes in the spleen during the course of infection. We find that per oral infection with virulent *Salmonella* causes splenomegaly and disruption of splenic architecture, much like reported here (unpublished observations). However, the rate at which these changes take place is slower when mice are infected per orally. The use of TEM and IFM image analysis allows for the preservation of tissue architecture and visualization of the in situ changes that take place during the course of infection, thus TEM and IFM analysis complements flow cytometry analysis. Attenuated *Salmonella* vaccines are being developed to target not only *Salmonella*, but also other pathogens via delivery of heterologous antigens [20, 21, 24]. However, there is a risk that even highly attenuated strains may cause tissue damage upon systemic spread, therefore care must be taken when choosing attenuation strategies. In addition, alterations of the splenic architecture described here can greatly impact the immunogenicity of *Salmonella* vaccine strains. In conclusion, these findings add to the understanding of *Salmonella* pathogenesis and will aid in the development of *Salmonella* vaccines and therapies.

## Supporting Information

S1 Fig *Salmonella* persists in the spleens of infected mice up to day 21 after i.v. infection with 1x10^5^ CFUs. (TIF)

S2 Fig Two color IFM images depicting pathologic splenic rupture in mice infected with 1x10^5^ CFUs of *Salmonella* at day 6 (A) and day 14 (B) after infection. (TIF)

S3 Fig TEM images of spleen tissues from control (uninfected) mice and mice infected with *Salmonella*. (A, C) In control spleens, lymphocytes (*) predominate and macrophages (Mϕ) are more compact and have smaller cytoplasms.
(TIF)

S4 Fig Representative three color images showing the expansion of F4/80^+^ macrophages and erythroid cells in the spleens of uninfected mice (control, day 0) and mice infected i.v. with 1x10^5^ CFUs of *Salmonella* at day 9, 14, and 21 after infection. (TIF)

S5 Fig TEM images of spleen sections of *Salmonella*-infected mice. (TIF)

S6 Fig Representative two color images showing B220^+^ lymphocytes in the spleens of uninfected mice (control, day 0) and mice infected i.v. with 1x10^5^ CFUs of *Salmonella* at day 6 and 21 after infection. (TIF)

S7 Fig Representative single-color and two-color confocal images showing colocalization of B220^+^ lymphocytes, CD4^+^ lymphocytes, and CD8^+^ lymphocytes with F4/80^+^ macrophages in spleens of mice infected with 1x10^5^ CFUs of *Salmonella*. (TIF)

S8 Fig TEM images of giant macrophages (Mϕ) in the spleens of S*almonella*-infected mice phagocytosing and “corralling” RBCs and lymphocytes (L). (TIF)
