# Supplementary figures and images for: Infection with Salmonella enterica Serovar Typhimurium Leads to Increased Proportions of F4/80+ Red Pulp Macrophages and Decreased Proportions of B and T Lymphocytes in the Spleen
Source: PLoS One. 2015 Jun 12;10(6):e0130092. doi: 10.1371/journal.pone.0130092 (PMC4466801; doi:10.1371/journal.pone.0130092)

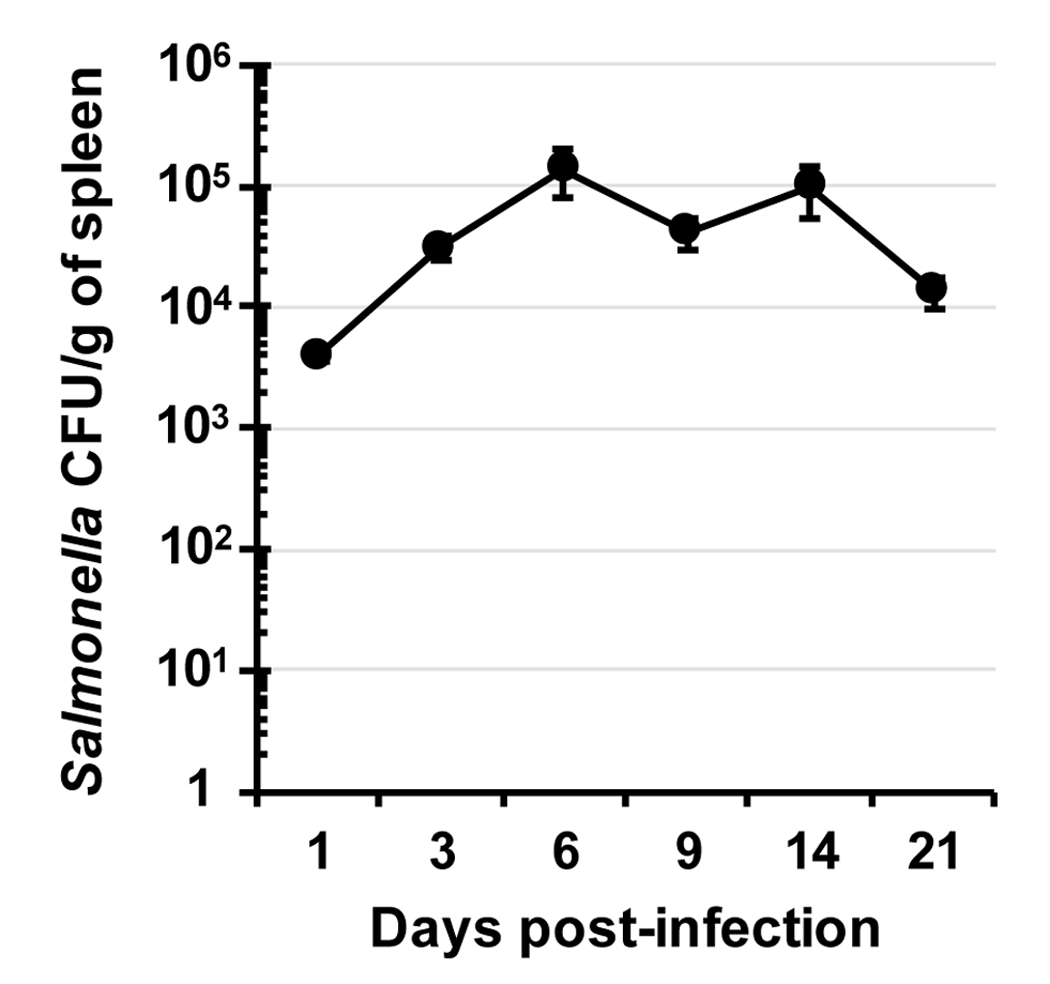

Supplement: S1 Fig — (TIF) [file pone.0130092.s001.tif]

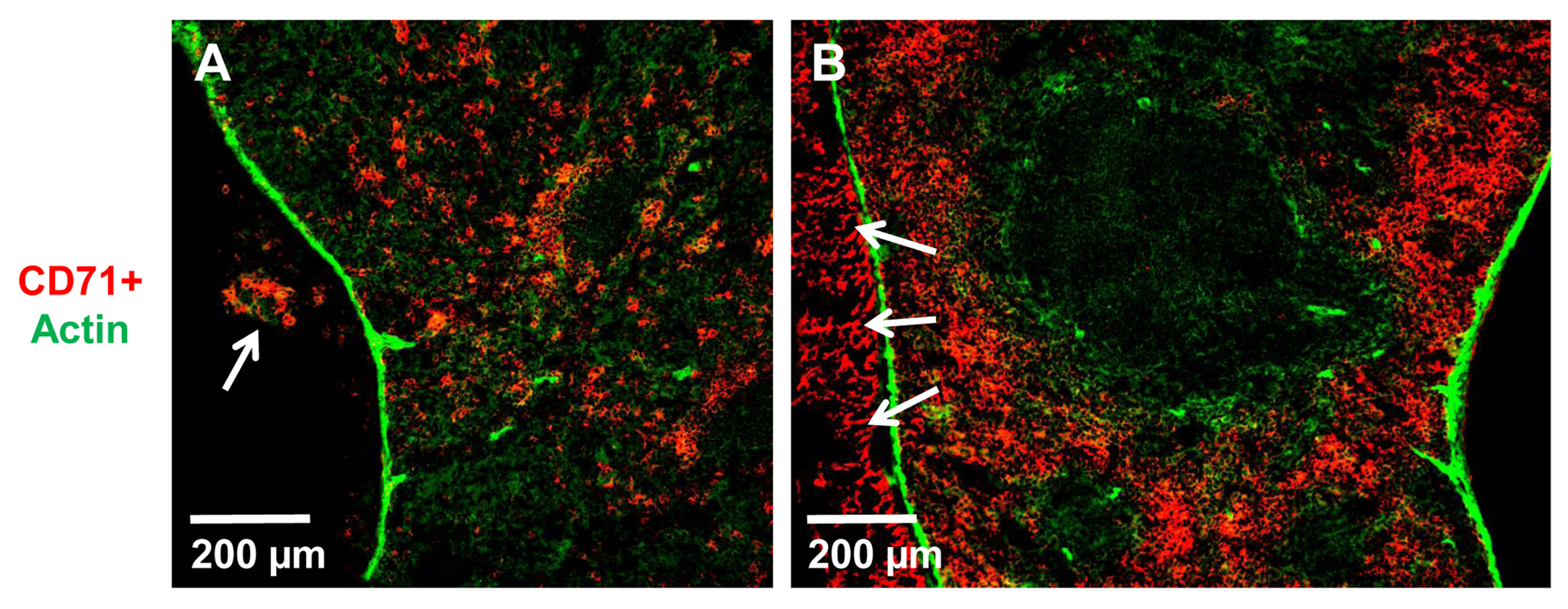

Supplement: S2 Fig — (TIF) [file pone.0130092.s002.tif]

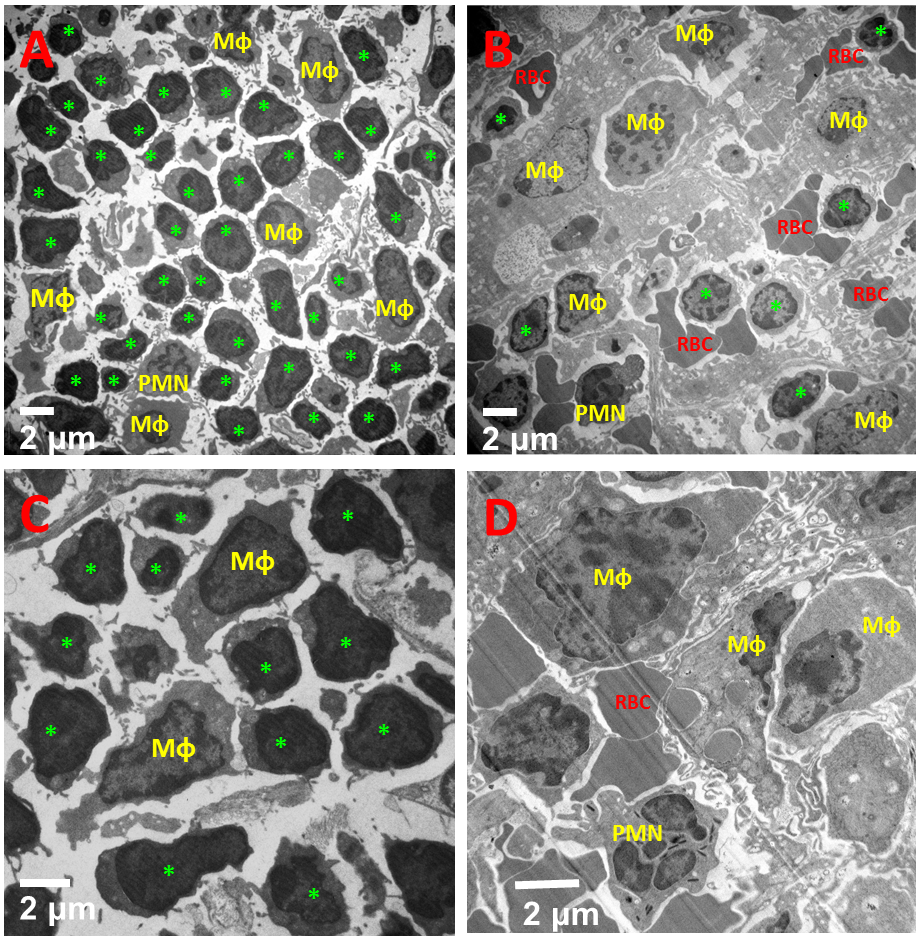

Supplement: S3 Fig — (A, C) In control spleens, lymphocytes (*) predominate and macrophages (Mϕ) are more compact and have smaller cytoplasms. (TIF) [file pone.0130092.s003.tif]

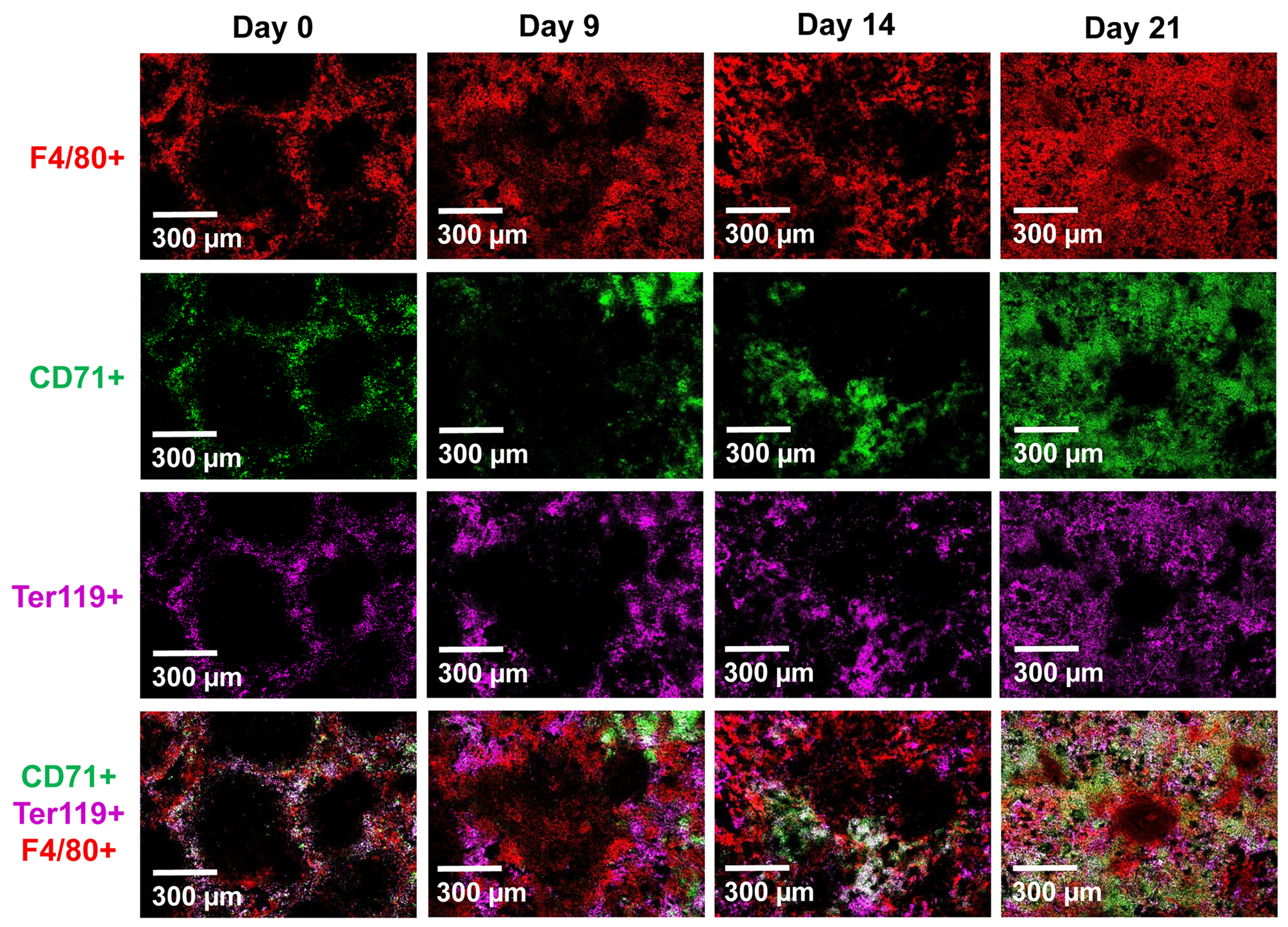

Supplement: S4 Fig — (TIF) [file pone.0130092.s004.tif]

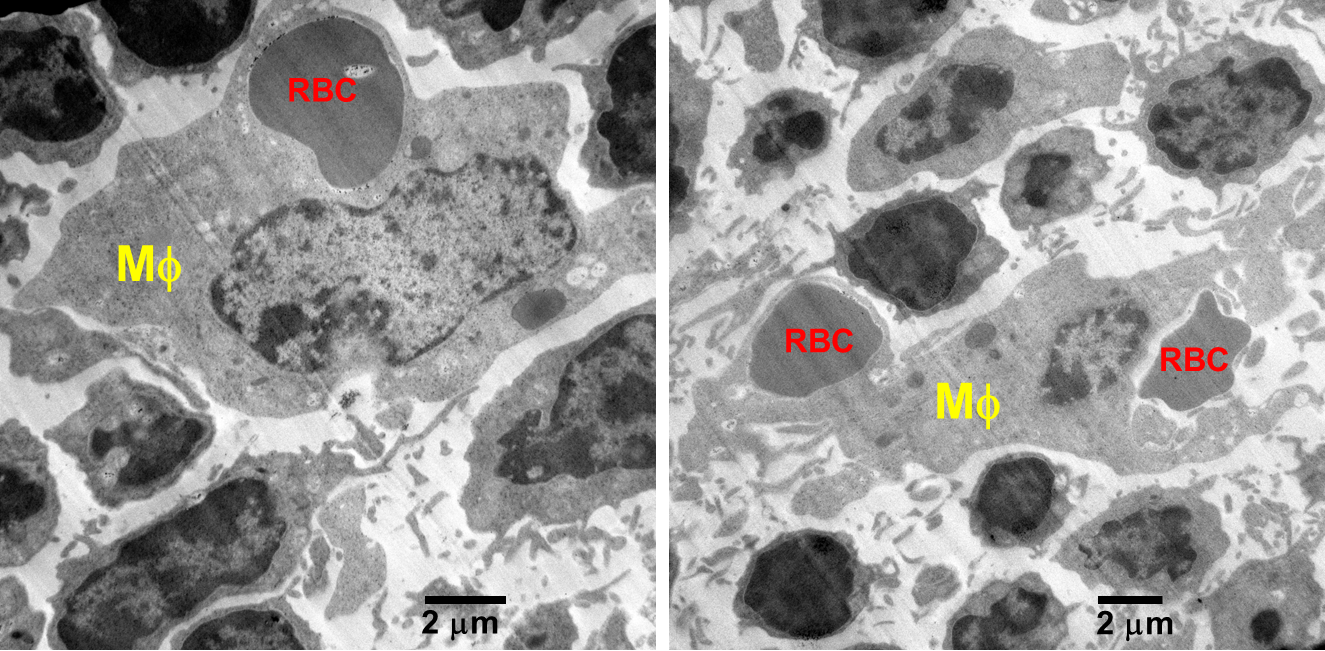

Supplement: S5 Fig — (TIF) [file pone.0130092.s005.tif]

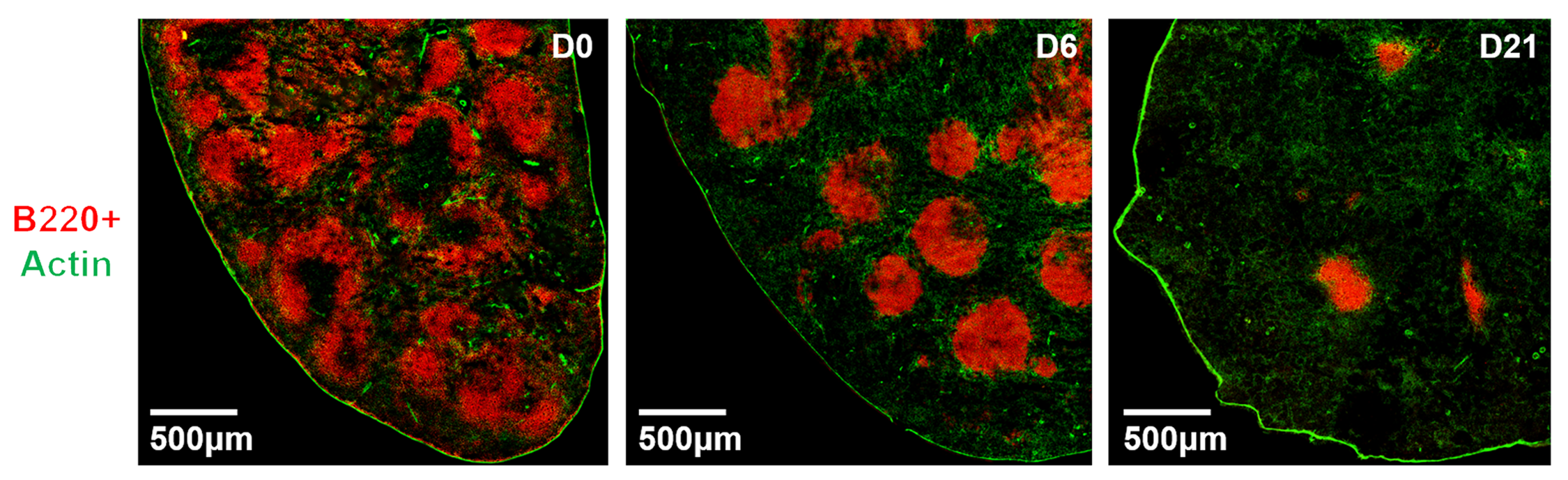

Supplement: S6 Fig — (TIF) [file pone.0130092.s006.tif]

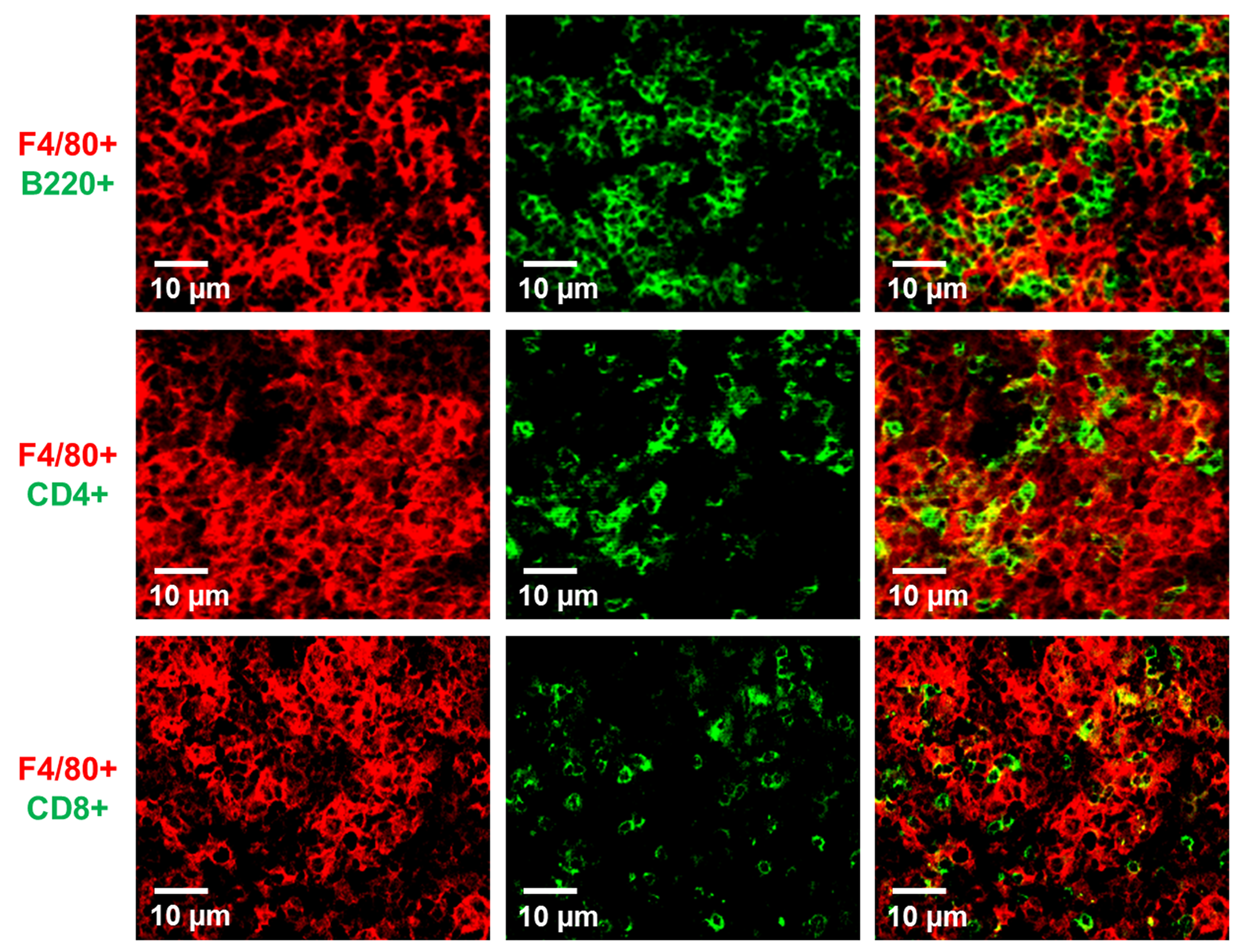

Supplement: S7 Fig — (TIF) [file pone.0130092.s007.tif]

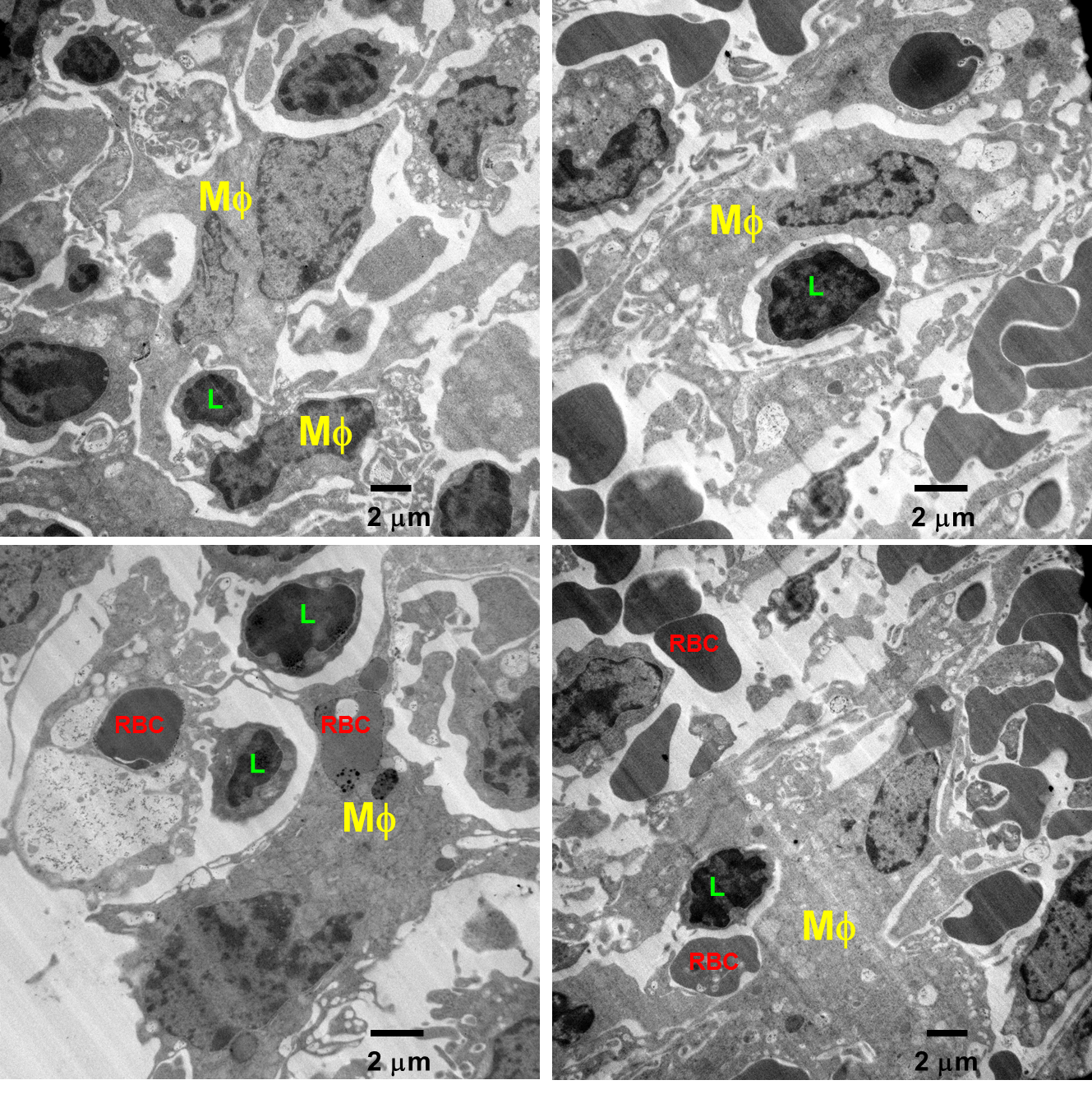

Supplement: S8 Fig — (TIF) [file pone.0130092.s008.tif]
